# Effects of BNP and Sacubitrilat/Valsartan on Atrial Functional Reserve and Arrhythmogenesis in Human Myocardium

**DOI:** 10.3389/fcvm.2022.859014

**Published:** 2022-07-05

**Authors:** Uwe Primessnig, Peter M. Deißler, Paulina Wakula, Khai Liem Tran, Felix Hohendanner, Dirk von Lewinski, Florian Blaschke, Christoph Knosalla, Volkmar Falk, Burkert Pieske, Herko Grubitzsch, Frank R. Heinzel

**Affiliations:** ^1^Department of Internal Medicine and Cardiology, Charité-Universitätsmedizin Berlin, Campus Virchow-Klinikum, Berlin, Germany; ^2^DZHK (German Centre for Cardiovascular Research), Berlin, Germany; ^3^Berlin Institute of Health (BIH), Berlin, Germany; ^4^Department of Cardiology, Medical University of Graz, Graz, Austria; ^5^Department of Cardiothoracic and Vascular Surgery, German Heart Institute Berlin, Berlin, Germany; ^6^Department of Cardiovascular Surgery, Charité-Universitätsmedizin Berlin, Corporate Member of Freie Universität Berlin, Humboldt-Universität Berlin, and Berlin Institute of Health, Berlin, Germany; ^7^Department of Internal Medicine and Cardiology, German Heart Center Berlin, Berlin, Germany

**Keywords:** BNP, sacubitrilat/valsartan (Sac/Val), atrial function, arrhythmias, heart failure, neprilysin, sacubitril/valsartan

## Abstract

**Background:**

Although the angiotensin receptor-neprilysin inhibitor (ARNI) sacubitril/valsartan started a new era in heart failure (HF) treatment, less is known about the tissue-level effects of the drug on the atrial myocardial functional reserve and arrhythmogenesis.

**Methods and Results:**

Right atrial (RA) biopsies were retrieved from patients (*n* = 42) undergoing open-heart surgery, and functional experiments were conducted in muscle strips (*n* = 101). B-type natriuretic peptide (BNP) did not modulate systolic developed force in human myocardium during β-adrenergic stimulation, but it significantly reduced diastolic tension (*p* < 0.01) and the probability of arrhythmias (*p* < 0.01). In addition, patient's plasma NTproBNP positively correlated with isoproterenol-induced contractile reserve in atrial tissue *in vitro* (*r* = 0.65; *p* < 0.01). Sacubitrilat+valsartan (Sac/Val) did not show positive inotropic effects on atrial trabeculae function but reduced arrhythmogeneity. Atrial and ventricular biopsies from patients with end-stage HF (*n* = 10) confirmed that neprilysin (NEP) is equally expressed in human atrial and ventricular myocardium. RA NEP expression correlates positively with RA ejection fraction (EF) (*r* = 0.806; *p* < 0.05) and left ventricle (LV) NEP correlates inversely with left atrial (LA) volume (*r* = −0.691; *p* < 0.05).

**Conclusion:**

BNP ameliorates diastolic tension during adrenergic stress in human atrial myocardium and may have positive long-term effects on the inotropic reserve. BNP and Sac/Val reduce atrial arrhythmogeneity during adrenergic stress *in vitro*. Myocardial NEP expression is downregulated with declining myocardial function, suggesting a compensatory mechanism in HF.

## Introduction

Heart failure (HF) is a major burden in Western societies, both economically and in terms of disability-adjusted life years lost. Its prevalence is increasing with age up to 10% in the 8th decade of life (1, 2). HF with reduced ejection fraction (EF) and HF with preserved EF are characterized by a reduced functional reserve to physiological stress, such as adrenergic stimulation (3). The heart's atria play an important role in HF, as they improve ventricular filling and contribute to ventricular stroke volume and cardiac output to up to 40% during periods of hemodynamic demand (4). Atrial dysfunction and remodeling are closely associated with new-onset HF, atrial fibrillation, and overall increased mortality (5–7). The atria also contribute to endocrine signaling in HF, especially by releasing natriuretic peptides (NPs), such as atrial natriuretic peptide (ANP) and B-type natriuretic peptide (BNP). NPs modulate the functional reserve of the ventricular myocardium; however, their role in modulating the atria's functional reserve is unknown (8).

One of the pivotal enzymes involved in NP degradation is neprilysin (NEP), an endopeptidase located on the extracellular portion of the cell membrane (9). The regulation of NEP expression in HF is part of an ongoing discussion, as an early study found NEP upregulation in human ventricular cardiomyocytes in patients with aortic stenosis and HF (10), while a more recent study in a porcine model of ischemic cardiomyopathy suggested a systemic downregulation of total NEP expression (11). The expression of NEP in human atrial tissue has not been reported yet.

In 2014, the PARADIGM-HF trial introduced the first of its kind angiotensin receptor-neprilysin inhibitor (ARNI), sacubitril/valsartan, which exerts its function by a dual mechanism, namely, angiotensin receptor blockage and NEP inhibition (12). Besides its beneficial effects on HF, evidence supports a reduced probability of (ventricular) arrhythmias in patients with HF on ARNI treatment (13–15). The exact mechanisms involved are unknown and supposedly multifactorial. By increasing the NP levels and stimulating the cyclic guanosine monophosphate/ protein kinase G (cGMP/PKG) pathway, sacubitrilat (Sac; the active metabolite of sacubitril) may improve cardiomyocyte function in (early) cardiac remodeling, as reported for ANP in a murine model of HF (8). Thus, we hypothesized that an increase in NP tissue concentration by Sac without or with angiotensin-receptor blockade by valsartan (Val) may exert beneficial effects on atrial myocardial function reserve and arrhythmias.

In this study, we, therefore, investigated the functional and antiarrhythmic effects of BNP and Sac without and with Val on human atrial myocardium. Furthermore, we also analyzed the expression of NEP in failing human left ventricular (LV) and right atrial (RA) myocardial samples.

## Methods

### Patient Selection

The study was performed in line with the 1975 Declaration of Helsinki ethical guidelines. Sample acquisition at Charité Campus Virchow-Klinikum and German Heart Center Berlin (DHZB) was approved by the Local Ethics Committee of the Charité (EA2/167/15) and the Ethics vote for the Biobank at the DHZB (EA4/028/12). All patients were ≥18 years old and provided written informed consent prior to enrolment. Patients with active malignancy, congenital heart disease, and endocarditis were not enrolled in our study, and one patient was excluded due to reported chronic right coronary artery occlusion. RA myocardial tissue and peripheral blood serum samples were obtained as part of the routine surgical procedure for patients undergoing heart surgery with extracorporeal circulation (predominantly coronary artery bypass graft or aortic valve replacement surgeries; see data specified below). Relevant medical and drug history was obtained from the patients' files.

### Analysis of Echocardiography Recordings

Transthoracic echocardiography was performed within a week before surgery by an experienced investigator, using an Epiq 7G station (Philips, Andover, MA, USA) with a 2.5-MHz probe, and loops were recorded using standardized protocols. Echo data sets on (preoperative) ventricular and atrial function were reviewed and reanalyzed as applicable regarding the relevant readouts by an independent examiner (U.P.). According to the local standard operating procedure, left atrial (LA) and RA speckle-tracking analysis was performed using TomTec software (TOMTEC Imaging Systems GmbH, Unterschleißheim, Germany).

### Functional Human Myocardial Experiments

Human myocardium for functional measurements was collected as excess tissue from the RA appendage (RAA) as part of the standard surgical procedure for patients undergoing open-heart surgery with extracorporeal circulation (aortic valve replacement surgeries and coronary artery bypass grafts). RAA biopsies from 42 patients with cumulative 101 muscle strips were included in our study collected between December 2018 and April 2020. Trabeculae were excised using microsurgical scissors and forceps. Human atrial muscle strips (≥3 mm length >0.65 mm, and preferred <1 mm diameter) were isolated, as the risk of central ischemia increases with muscle diameter. Directly after the end of the functional measurements, all muscle strips were snap-frozen in liquid nitrogen and stored for further molecular biology analysis. Functional atrial myocardium measurements were performed with a force transducer that recorded every muscle contraction (developed force) and relaxation. Muscle strips were stimulated at 1 Hz and 5 ms rectangular field stimulation and then prestretched up to the point of maximal force development (L_max_), as also described previously (16). The acquisition was performed using the software MyoDat (MyoTronic UG, Heidelberg, Germany). Analysis of systolic and diastolic functional parameters was performed using the program MyoViewer (MyoTronic UG, Heidelberg, Germany). Furthermore, the incidence of arrhythmias (=spontaneous aftercontractions) was determined (see Supplementary Material for detailed information).

### Experimental Protocols and Pharmacological Interventions

We investigated the effects of BNP, Sac (the active metabolite of sacubitril), and the combination of Sac with Val (Sac/Val) on atrial functional reserve and arrhythmogenesis in the presence of physiological stressors and varying stimulation frequency (1, 2, 3, and 0.5 Hz) in 42 patients (*n* = 101 muscle strips). Atrial trabecula were all treated with the physiological stressor isoproterenol (ISO), which is an unspecific beta-adrenoreceptor (β-AR) agonist. A concentration of 20 nM was used, as this marks a half-maximal saturation of the protein kinase A (PKA)-mediated phosphorylation (17). To investigate the effect of BNP on atrial trabeculae, the intervention consisted of treatment with 100 nM BNP, which was found to be the dose of maximal BNP effect in a study conducted by Guo et al. (18). Furthermore, the effect of either 8.5 μg/ml Sac or the combination of Sac with 4.0 μg/ml Val was investigated on atrial functional reserve or arrhythmogenesis. Those concentrations were found to be the peak plasma concentrations of Sac/Val following oral treatment with an ARNI (19, 20). Finally, after the addition of BNP, Sac, or Sac/Val, different stimulation frequencies were tested beginning at 1 Hz and increasing to 2 and 3 Hz, followed by 0.5 Hz in the end.

### Western Blot VASP-Ser239/-Ser157 Phosphorylation

Vasodilator-stimulated phosphoprotein (VASP)-Ser239 and -Ser157 phosphorylation was investigated in human atrial muscle strips (control strips and strips treated with Sac/Val) using Western blot analysis (see Supplementary Material for detailed information).

### Neprilysin Enzyme-Linked Immunosorbent Assay

NEP levels were determined in LV and RA biopsies from patients (*n* = 10) with end-stage HF. Tissue was analyzed by the enzyme-linked immunosorbent assay (ELISA) kit (EHMME, Thermo Scientific, USA) according to the manufacturer's instruction. The total protein concentration in supernatants was determined *via* Pierce BCA assay (Thermo Scientific). Samples were then diluted in the ratio of 1:5 and assessed in duplicate. The concentration (ng/ml) was normalized to total protein content (ng/mg of total protein). See Supplementary Material for detailed information.

### Statistical Analysis

**Figure 1:**
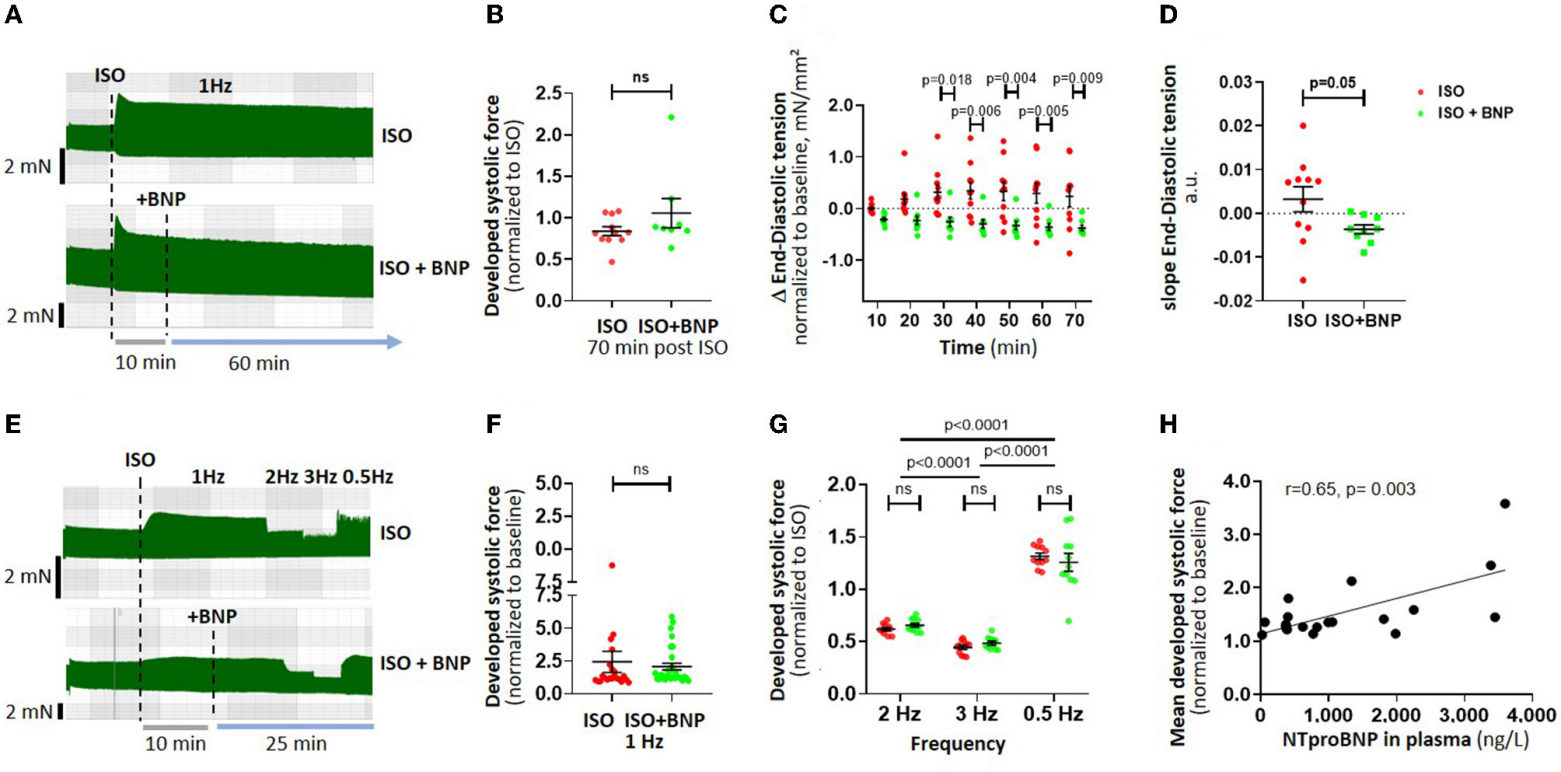
Effect of BNP on atrial inotropy and lusitropy after adrenergic stimulation. Example traces of time-dependent analysis of BNP (*n* = 20 strips) are shown in **(A)**, with the corresponding data on the effect of BNP treatment on atrial systolic force **(B)**, and end-diastolic tension generation **(C)** after 70 min. The slope of end-diastolic tension over time is shown in **(D)**. The effects of a frequency-variation protocol [example traces in **(E)**, *n* = 40 strips] on systolic force at different stimulation frequencies are shown in **(F,G)**. Correlation of patient plasma NTproBNP and relative force increase after ISO treatment (values from *n* = 19 patients with available NTproBNP) is shown in **(H)**. Data are shown as mean ± SEM; each data point represents one muscle strip. In **(H)**, each point represents the mean developed systolic force per patient. ISO, isoproterenol; BNP, brain natriuretic peptide.

**Figure 2:**
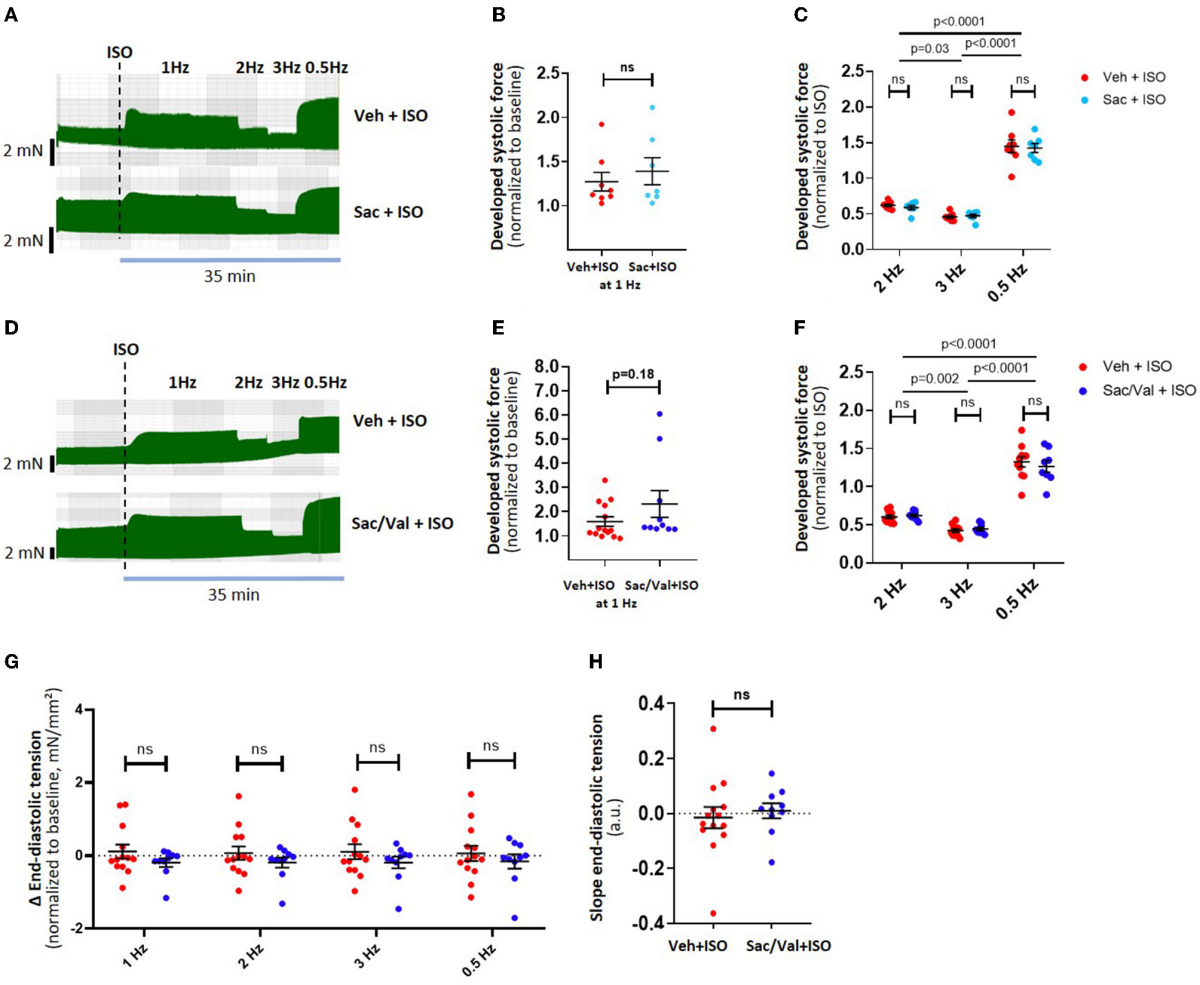
Effect of Sac/Val on atrial inotropy and lusitropy after adrenergic stimulation. Example traces of a “frequency-variation protocol” in Sac-treated muscle strips (*n* = 15 strips) are shown in **(A)**, with its effects on developed force during different frequencies shown in **(B,C)**. Examples traces of a frequency-variation protocol in Sac/Val treated muscle strips (*n* = 24 strips) are shown in **(D)**, with the corresponding effects on developed force in **(E,F)**. The effects of Sac/Val on end-diastolic tension are shown as per stimulation frequency **(G)** and as the diastolic tension slope derived from linear regression **(H)**. Data are shown as mean ± SEM; each data point represents one muscle strip. ISO, isoproterenol, Sac: Sacubitrilat; Val: valsartan; Veh: vehicle (DMSO).

**Figure 3:**
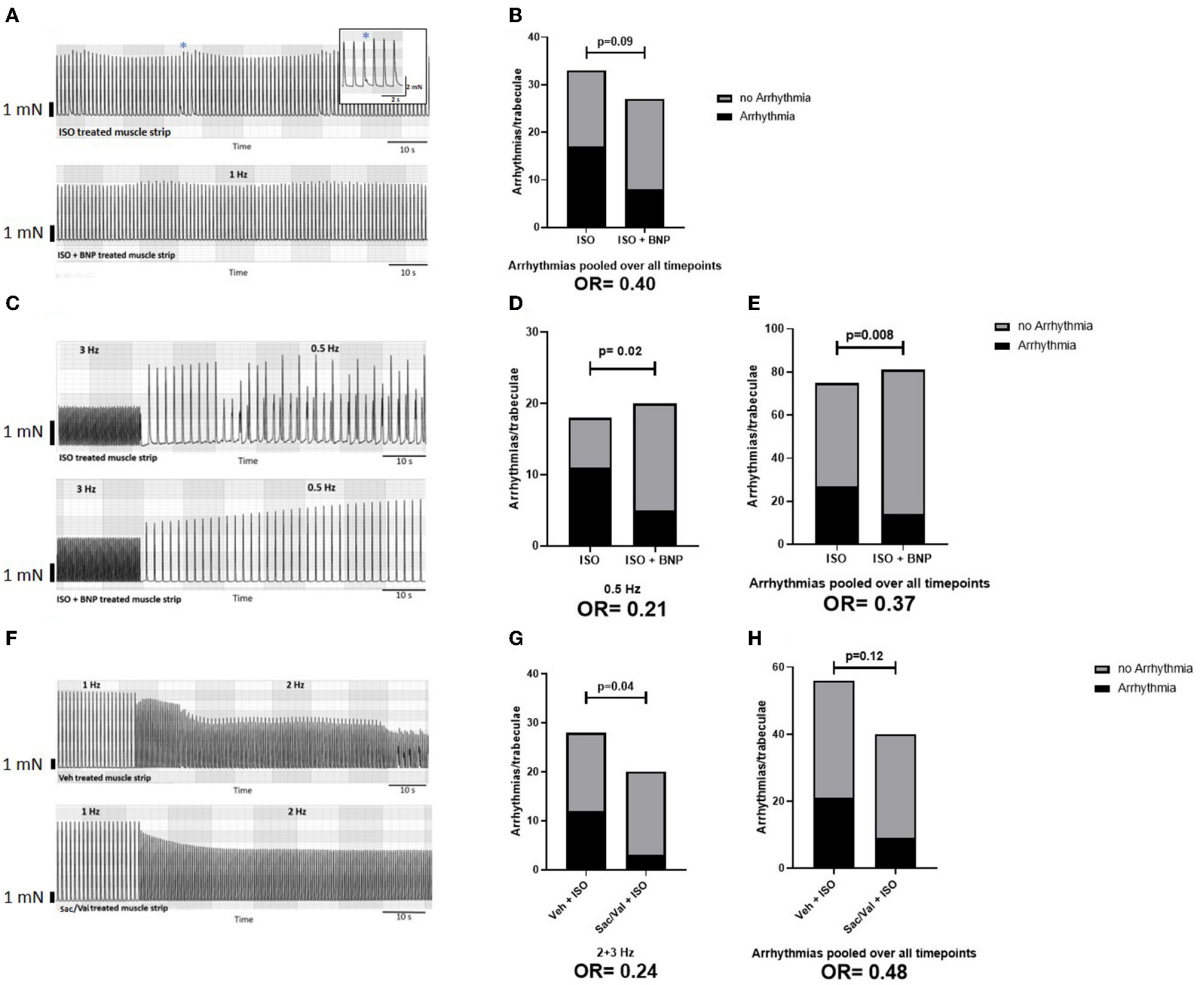
Effect of BNP and Sac/Val on atrial arrhythmogenesis. **(A)** Representative traces of a protocol with constant stimulation at 1 Hz in strips treated with ISO ± BNP; the overall effect of BNP treatment on arrhythmogenesis in this protocol is shown in **(B)**. For this analysis, three time intervals (25–30, 45–50, and 65–70 min) after ISO have been screened for arrhythmias. **(C)** Example traces for muscle strips treated with ISO ± BNP during the switch from 3 Hz to 0.5 Hz stimulation frequency in a “frequency-variation protocol” protocol. The effects of BNP on arrhythmogenesis at 0.5 Hz **(D)** and pooled over all time points **(E)** are shown. For this analysis, the arrhythmias at 1 Hz (10 min) and 2, 3, and 0.5 Hz (each 5 min) have been included Finally, example traces of muscle strips treated with a vehicle (DMSO) or Sac/Val at the transition between 1 and 2 Hz stimulation frequency are shown in **(F)**, with the effects of Sac/Val treatment on arrhythmias at higher stimulation frequencies in **(G)** and the overall effect on arrhythmia in **(H)**. For this analysis, the arrhythmias at 1 Hz (10 min) and 2, 3, and 0.5 Hz (each 5 min) have been included. ISO, isoproterenol; BNP, brain natriuretic peptide; Veh, vehicle (DSMO); Sac, sacubitrilat; Val, valsartan.

**Figure 4:**
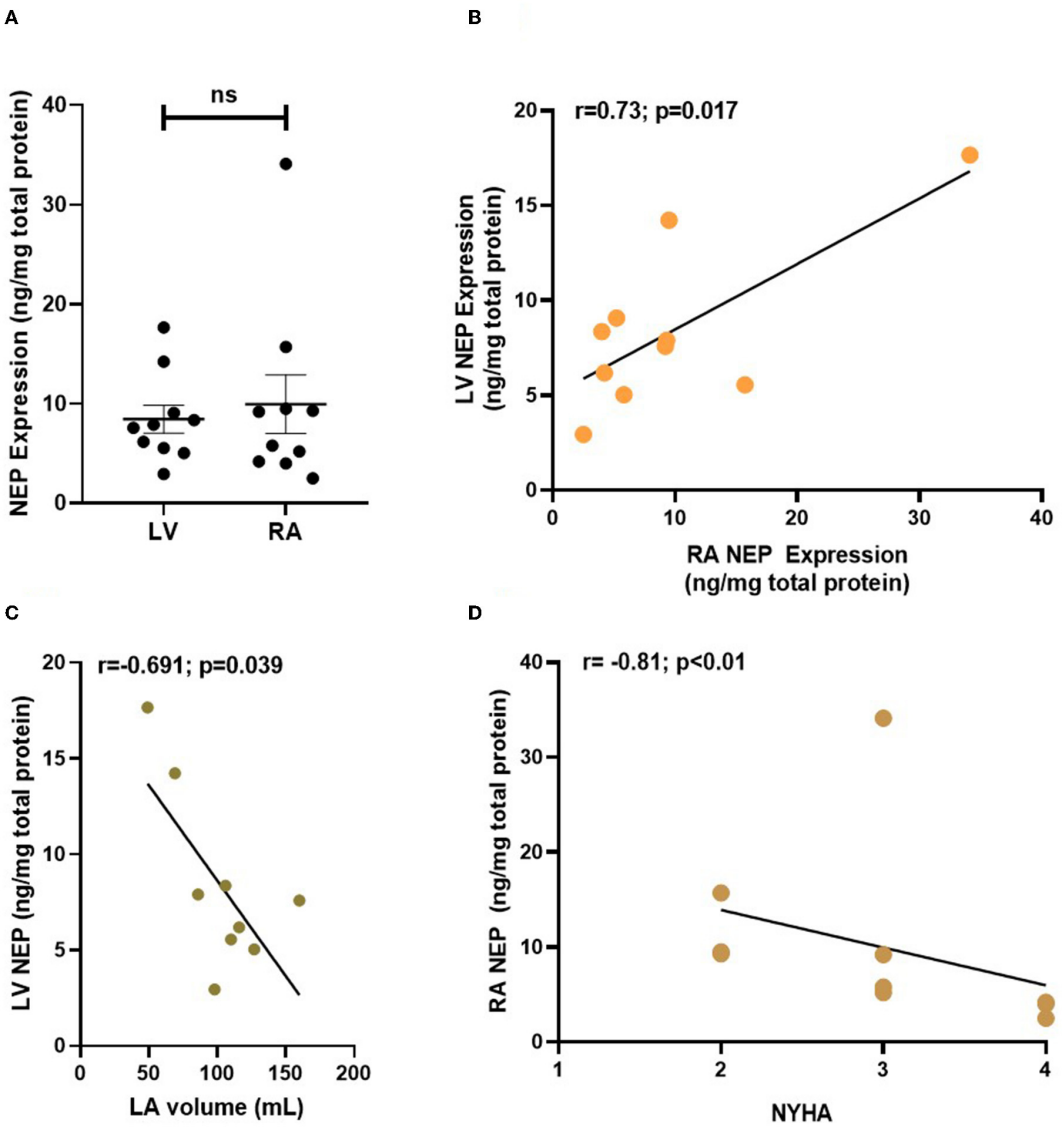
NEP expression (in ng/mg of total protein) in human atrial and ventricular samples from patients with end-stage heart failure. NEP expression was analyzed in RA and LV biopsies from *n* = 10 patients with end-stage heart failure. NEP is equally expressed in the failing atrial and ventricular myocardium **(A)** and expression of atrial and ventricular NEP correlates in the patient cohort **(B)**. LV NEP expression correlates inversely with LA volume **(C)** and RA NEP negatively correlates with patient NYHA **(D)**. Each point represents data from one patient. NEP, Neprilysin; LV, left ventricle; LA, left atrium; RA, right atrium.

Statistical analysis and figure design were performed using GraphPad Prism version 8.0.1. Data points in the graphs represent single muscle strip experiments, and error bars are presented as the standard error of means (S.E.M) if not stated otherwise. Multiple comparisons of functional parameters (developed systolic force and diastolic tension) were analyzed with a two-way analysis of variance (ANOVA) with repeated measures (RMs) and Sidak *post-hoc* test (Figures 1C,G, 2C,F,G; Supplementary Figures 1A, 2). Comparison of two groups was done by unpaired two-sided *t*-test (Figures 1B,D,F, 2B,E,H, **4A**; Supplementary Figures 1B, 3A,B), and arrhythmias were quantified by the two-sided chi-square test and additional calculation of odds ratio by the Baptista-Pike method (Figures 3A–H). Correlations were analyzed by either Pearson *r* (Figures 1H, 4B,C; Supplementary Figure 4) or Spearman *r* test (Figure 4D). For all analyses, a two-sided *p*-value of *p* < 0.05 was considered to be significant.

## Results

### Patient Characteristics and Muscle Strip Experiments

Overall, 42 patients were included in our study between December 2018 and April 2020 for functional measurements in atrial muscle strips (*n* = 101). Most patients suffered from at least one cardiovascular risk factor (Table 1A). Treatment included angiotensin-converting-enzyme (ACE) inhibitors/AT1 blockers blockers and beta-blockers in most patients. The biomarker analysis showed normal blood count and electrolytes, elevated NTproBNP levels, and, on median, a slightly reduced glomerular filtration rate (GFR) (Table 1A). The echocardiographic evaluation demonstrated a slightly reduced LVEF with a median of 53% overall. LV hypertrophy and mild diastolic dysfunction were observed in most patients (Table 1B).

**Table 1:** Patient characteristics.

**(A)**						
**Patient characteristics**			**Medication**			
Age	71 (66–77)		ACE Inhibitors/ AT1–RB	76 % 32/42		
Sex (male)	86 % 36/42		Beta–Blocker	64 % 27/42		
BMI	27 (24–30)		Diuretics	36 % 15/42		
Arterial hypertension	76 % 32/42		MRI	14 % 6/42		
Dyslipidemia	45% 19/42		ARNI	2 % 1/42		
Adipositas	26 % 11/42		Statine	64 % 27/42		
Diabetes mellitus	33 % 14/42		Oral antidiabetic	26 % 11/42		
Coronary artery disease	88 % 37/42		Insulin	5 % 2/42		
Atrial Fibrillation	26 % 11/42					
COPD	14 % 6/42		**Type of surgery**			
Heart failure	29 % 12/42		Coronary Artery Bypass Graft	83 % 35/42		
Chronic kidney disease	17 % 7/42		Aortic Valve Replacement or reconstruction	24 % 10/42		
Smoker	33 % 14/42		Mitral Valve Replacement or reconstruction	5 % 2/42		
Alcoholism	7 % 3/42		Aortic–Root–Replacement	12 % 5/42		
**Laboratory markers**						
Hb (g/dl)	14 (13–15)		N = 42			
Na+ (mmol/l)	140 (138–143)		N = 42			
K+ (mmol/l)	4.2 (4.0–4.5)		N = 42			
NT–proBNP (ng/l)	769 (396–1984)		N = 23			
GFR (ml/min)	76 (61–90)		N = 42			
Creatinin (mg/dl)	0.95 (0.79–1.17)		N = 42			
CRP (mg/l)	2.75 (0.9–11.23)		N = 42			
HbA1c (mmol/mol)	41 (38–46)		N = 19			
**(B)**						
**Echo data**						
LVEF (%)	53 (45–57)		N = 42			
LVEDD (mm)	46 (44–52)		N = 41			
IVSd (mm)	13 (12–14)		N = 41			
PWd (mm)	12 (11–12)		N = 41			
LA volume biplan (ml)	61 (51–81)		N = 36			
LAVI (ml/m2)	31 (27–38)		N = 35			
LA diameter (mm)	38 (35–40)		N = 39			
LA emptying fraction (norm >37%)	50 (35–55)		N = 35			
LA strain (>23% norm)	19 (14–29)		N = 20			
RA area (cm2)	16 (14–20)		N = 36			
RA diameter (mm)	35 (28–38)		N = 38			
RA emptying fraction (norm >37%)	52 (33–59)		N = 32			
RA strain reservoir+conduit (ϵe)	42 (31–49)		N = 14			
E/É	11 (10–13)		N = 17			
TAPSE (mm)	19 (17–23)		N = 39			
RVEDD (mm)	35 (31–39)		N = 37			
sPAP (mmHg)	25 (24–31)		N = 21			

*Clinical characteristics of patient cohort included for in vitro experiments **(A)** and echo characterization of patient cohort **(B)**. Data are presented as “median (IQR)” or “percent (n patients/all patients).” MRI, mineralocorticoid receptor inhibitor; ARNI, angiotensin receptor-neprilysin inhibitor; LV, left ventricle; LA, left atrium; RA, right atrium; AT1-RB, Angiotensin II receptor blocker*.

### Acute Effect of BNP on Atrial Inotropy and Lusitropy

We tested the time-depended effect of BNP (100 nM) during adrenergic stimulation with 20 nM ISO over 70 min on diastolic tension and developed systolic force (*n* = 20 strips from *N* = 9 patients, Figure 1A). BNP did not significantly increase systolic developed force in response to prolonged ISO treatment (70 min, 1 Hz stimulation, Figure 1B). BNP significantly lowered diastolic tension in ISO-treated atrial muscle strips (overall *p* = 0.005, two-way ANOVA with RM, Figure 1C), as also reflected by a higher negative slope in end-diastolic tension over time (Figure 1D).

Furthermore, we investigated the effects of BNP on frequency-dependent modulation of the functional response to ISO (Figure 1E, *n* = 40 muscle strips from 17 patients). Developed systolic force in the presence of ISO decreased with higher frequency stimulation (2 and 3 Hz) and recovered with 0.5 Hz (Figures 1E–G). Treatment with BNP did not alter the frequency-dependent atrial systolic force response or contraction kinetics at any frequency (Figures 1F,G). At 3 and 0.5 Hz, BNP furthermore significantly reduced end-diastolic tension (ISO+BNP vs. ISO: 3 Hz <0.05; 0.5 Hz: *p* < 0.01, overall *p* = 0.01, two-way ANOVA with RM) (Supplementary Figure 1A). The slope of end-diastolic tension during the frequency stair was positive in ISO but slightly negative in ISO+BNP (Supplementary Figure 1B, *p* = 0.09).

We found that the mean inotropic reserve of atrial muscle strips in response to isoproterenol *in vitro* correlated well with preoperative plasma NTproBNP values of the same patient (available from *n* = 19 patients without preincubation, Figure 1H).

### Effect of Sac/Val on Atrial Inotropy and Lusitropy

We evaluated the effects of the NEP inhibitor Sac on the atrial functional reserve. Preincubation (1 h) and the presence of Sac alone did not affect the developed force or diastolic tension after ISO at different stimulation frequencies (Figures 2A–C, *n* = 8 and 7 muscle strips, respectively, from 5 patients). Preincubation (1 h) and the presence of Sac in combination with Val in concentrations matching the peak plasma levels found in patients treated orally with an ARNI showed a slight trend toward an increased inotropy with ISO compared to control (Figures 2D–F) at 1 Hz, but otherwise had no significant effects on developed force or kinetics (time to peak, half time of relaxation, or relaxation constant tau; data not shown). Interestingly, despite the effects seen with BNP earlier, diastolic tension was unchanged with Sac/Val (Figures 2G,H; *n* = 24 muscle strips from 9 patients, overall *p* = 0.3, two-way ANOVA with RM).

### Effect of BNP and Sac/Val on Atrial Arrhythmias During Cardiac Stress

During ISO treatment, some of the atrial muscle strips developed arrhythmias (aftercontractions, Figure 3A). In the protocol with prolonged ISO-treatment at 1 Hz, the addition of BNP tended to lower the incidence of arrhythmias (Figure 3B, *n* = 20 trabeculae from 9 patients). The antiarrhythmic effect of BNP was more pronounced in atrial muscle strips exposed to ISO and frequency stair (*n* = 40 trabeculae from 17 patients), with a pronounced reduction of arrhythmias in the recovery phase (0.5 Hz) following 3 Hz stimulation (Figures 3C,D). Overall the incidence of arrhythmias was reduced by 48% with BNP compared to the control group in this protocol [*p* = 0.008, two-sided chi-square test; odds ratio (OR) = 0.37; Figure 3E].

Sac/Val preincubation and presence tended to decrease overall arrhythmia burden during ISO and frequency stair (*p* = 0.12, two-sided chi-square test; OR = 0.48; Figures 3F–H) with a significant reduction of arrhythmias at higher stimulation frequencies (Figure 3G; *p* = 0.04, two-sided chi-square test; OR = 0.24, *n* = 24 trabeculae from 9 patients).

Sac treatment without Val had no impact on the prevalence of arrhythmias during adrenergic and frequency-dependent atrial stress (in *n* = 15 trabeculae from 5 patients, data not shown).

### Effects of Sac/Val on VASP Phosphorylation

We probed signaling downstream of cGMP by measuring phosphorylation of VASP as a surrogate of PKG activity (21). pVASP-Ser239 is a known indicator for (A)NP-dependent increase in phosphorylation (22). In a subset of muscle strips (*n* = 15 from 5 patients) treated with either Sac/Val or vehicle (dimethyl sulfoxide, DMSO) and undergoing the frequency protocol, VASP phosphorylation at Ser239 and Ser157 was studied. We did not observe any difference in VASP phosphorylation between Sac/Val-treated atrial muscle strips and control group (Supplementary Figure 3).

### Neprilysin Expression in Human End-Stage HF

NEP protein expression was measured in RA biopsies from patients with end-stage HF (*n* = 10, median LVEF 20%) that either underwent heart transplantation (70%) or left ventricular assistant device implantation (30%). We also measured NEP in the LV of these patients. The detailed patient characteristics can be found in Supplementary Table 1. Plasma NTproBNP was elevated to >6,500 pg/ml on average as a marker of severe end-stage HF. NEP was equally expressed in RA and LV tissue (Figure 4A), with a significant positive correlation of RA and LV NEP expression within the same patient (Figure 4B), and RA NEP expression and RA EF (Supplementary Figure 4). Interestingly, also a strong negative correlation was found between LA volume and LV NEP (Figure 4C), and between patients' NYHA class and RA NEP (*r* = −0.81; *p* < 0.01, Spearman *r* test; Figure 4D).

## Discussion

This study investigated the effects of BNP and Sac/Val on the functional reserve of human atrial myocardium. To the best of our knowledge, this is the first study to show that (i) BNP ameliorates increased diastolic tension during adrenergic stress and alleviates stress-induced atrial arrhythmogeneity *in vitro*; (ii) NEP is equally present in human atrial and ventricular myocardium, but its expression is reduced with a progression of cardiac dysfunction; and (iii) the combination of Sac and Val does not influence the adrenergic functional reserve in isolated atrial muscle but reduces arrhythmias in response to adrenergic stimulation.

### NEP Expression and Regulation

Even though ARNIs have been the subject of intense research during the past decade, only little attention has been paid to the expression of NEP in the failing human heart. Fielitz et al. (10) were the first to show that NEP expression and activity were altered in LV myocardium in HF. They reported an increase in NEP expression and activity in LV samples from patients with HF, which they suggested to contribute to increased local degradation of bradykinin and NPs (10). In contrast to that, Pavo et al. found a reduction in LV NEP expression, concentration, and activity in a porcine model of ischemic cardiomyopathy. They suggested that NEP downregulation might represent a counterregulatory mechanism to HF (11). In this study, we show NEP expression in human atrial myocardium in similar quantities as in human failing LV. The inverse correlation of atrial dilatation with LV NEP protein expression suggests that chronically increased cardiac pressures as reflected by atrial dilatation may be a trigger for NEP downregulation in the human heart. Indeed, NEP inhibition with candoxatril has been shown to reduce ventricular filling pressures (cardiac preload) (23, 24). Thus, downregulation of NEP may contribute to a compensatory reduction of preload in situations of increased wall strain in HF. However, candoxatril, in contrast, also increased systolic blood pressure, which is presumably due to increased angiotensin II and endothelin levels (23, 25). These effects diminish the positive effects of isolated NEP downregulation or inhibition (26, 27). The elevated levels of endothelin 1 or angiotensin II (both of which are synthesized within the myocardium, among others) also could explain why isolated NEP inhibition by Sac alone did not have any effects on functional parameters or arrhythmia in our *in vitro* study. The previously described mechanism of NEP downregulation in HF, therefore, may be, just like other compensatory mechanisms in HF [e.g., myocardial hypertrophy (28) and renin-angiotensin-aldosterone system (RAAS) activation (29)], considered to be at least partly maladaptive. Not until NEP inhibition is combined with angiotensin II receptor blockage, the full potential of this mechanism can be therapeutically exploited.

### BNP and Sac/Val Effect on Developed Systolic Force and Diastolic Tension

The ARNI treatment is accompanied by a significant increase in circulating BNP, as its degradation is partly inhibited (30). Gu et al. (19) could show that ANP levels are already increased as short as 15 min after oral administration of an ARNI in a rat model. In combination with other studies showing that ARNI treatment increased the EF in patients with HFrEF (31), this raised the question if there are also short-term effects of NPs and NEP inhibition on the systolic or diastolic functional reserve in human atrial myocardium.

Perera et al. demonstrated that ANP increases contractility in mice ventricular cardiomyocytes in a phase of early cardiac hypertrophy already seconds after wash-in, but only if the cells were pretreated with ISO. This positive inotropic effect was explained by an augmentation of cAMP signaling in the hypertrophied myocardium exerted by a spatial redistribution of cGMP sensitive PDE2 and PDE3 (8). In this study, we did not observe an immediate positive inotropic effect of BNP in human atrial myocardium. The first reason for the lack of immediate positive inotropic effects may be that our patient cohort was rather suffering from chronic than acute conditions (CAD in 91%, aHT in 81%, NTproBNP 769 ng/L); LV hypertrophy was found to be in some extent (IVSd median diameter 13 mm), but RA diameter (median 34 mm) did not indicate atrial enlargement. These findings are also in line with other publications studying coronary artery bypass graft (CABG) patient characteristics and echocardiography (32, 33). As chronic HF is associated with a reduction in NP-A receptor sensitivity, immediate natriuretic signaling may have been attenuated in the patient group presented in this study (34, 35). Furthermore, the positive-inotropic spatial redistribution of PDE2 and PDE3 between β-ARs was only described in the early stages of hypertrophy by Perera et al. (8). A loss of this early compensatory mechanism during progression/duration of cardiac hypertrophy due to NP and β-AR desensitization and/or phosphodiesterases (PDE) reorganization is very likely and can also explain the observed loss of positive inotropic effects of NPs in chronic stages of cardiac disease (as seen in our patient cohort) (8). Finally, differences between atrial and ventricular adaptive mechanisms may also contribute to the observations made: it is unclear to the extent in which the mechanism of PDE reorganization can also be found in atrial tissue, but our results suggest that, at least in chronic LV hypertrophy, atrial tissue does not exhibit cellular adaptive processes associated with increased inotropy after NP incubation. Thus, patient and sample characteristics could explain why these effects were not seen in the present cohort. It is imaginable that BNP does not show these effects at an equivalent dose to ANP. However, as both NPs were administered in a concentration of 100 nM, also in accordance with previous experimental findings (18), and as ANP and BNP bind to the same receptors (NPR-As), this was not further explored in this study.

Interestingly, with the addition of BNP, we did observe a time- and frequency-dependent reduction of diastolic tension during adrenergic stress (ISO), thus establishing BNP's effectiveness in improving lusitropy in isolated atrial myocardium after ISO treatment.

Sac/Val inhibits the degradation of NPs in the myocardium and, therefore, increases the tissue concentration of ANP and BNP. In isolated atrial tissue as used in our study, however, Sac/Val did not reproduce the effect of supramaximal BNP treatment. We, therefore, conclude that the BNP levels intrinsically recruitable (by stretch and adrenergic stimulation) are not sufficient to see an acute effect of Sac/Val treatment on diastolic atrial tension.

Interestingly, we observed a strong correlation between plasma NTproBNP levels and the adrenergic atrial functional reserve *in vitro*, which suggests that long-term effects of (B)NP on adrenergic signaling may play a role in modulating the atrial myocardial functional reserve. Especially, the interplay of cAMP- and cGMP regulated by PDEs, such as PDE3, may serve as an explanation for this effect, as PDE3 degrades up to 50% of cellular cAMP, but can be inhibited by cGMP (cGMP inhibited cAMP PDE) (36). Constantly elevated tissue BNP and cGMP levels would, therefore, inhibit the degradation of cAMP and strengthen the intracellular pathway mediated by ISO, ultimately leading to a rise in relative force increase as a long-term effect.

### BNP and LBQ/Val Effect on Atrial Arrhythmias

The PARADIGM-HF study did not only show a reduced risk of HF-related hospitalization and death in ARNI treatment but also a significant reduction in mortality by sudden cardiac death (SCD) (12, 37). For that reason, only recently the focus of ANRI research also shifted to study the effects of ARNIs on cardiac (ventricular) arrhythmias. de Diego et al. (31) published one of the first clinical studies assessing the influence of ARNI treatment on arrhythmia. While episodes of premature ventricular contractions (PVCs), non-sustained ventricular tachycardia (NSVT), and sustained ventricular tachycardia were significantly reduced in ARNI treatment, there was only a statistical trend in the reduction of atrial fibrillation episodes reported (*p* = 0.07; ns) (31). These findings were verified in another study with a 12-month follow-up (15).

Despite that, observational clinical data also suggested that ARNI treatment may lower the risk for the development of (recurring) atrial fibrillation and lower the disease burden and frequency of arrhythmic events in patients with non-permanent atrial fibrillation (38–41).

Interestingly, in our study, we could demonstrate that while Sac/Val did not have a significant influence on diastolic tension and, therefore, wall strain, it still reduced the probability of arrhythmias significantly in ISO-treated muscle strips. This suggests that the reduction of atrial arrhythmias is not only related to a reduction in strain (diastolic tension). In ventricular tissue, it has been shown that ANP (10 nM) significantly suppresses ISO-induced Ca^2+^ spark frequency (CaSF) and reactive oxygen species (ROS) production on a cellular level (42). Eiringhaus et al. demonstrated that in ventricular myocardium, Sac/Val decreases diastolic SR Ca^2+^ leak and CaSF. These effects were not visible under basal conditions but only after ISO treatment (43). Interestingly, in atrial myocardium, we could reproduce the antiarrhythmic effect at a nearly 50% lower Sac/Val dosage than Eiringhaus et al. (40 μmol). The dosage we used matched the peak plasma concentration achieved in humans in Sac/Val treatment (≈22.17 μmol Sac) (19, 20). That indicates that the antiarrhythmic properties of Sac/Val are exerted at a dose attained in standard HF treatment. As Sac and Sac/Val did not affect developed force in atrial muscle but external BNP reproduced the reduction in atrial arrhythmia incidence, we propose that the increased (juxtacellular) concentration of NPs by NEP-inhibitor Sac/Val contributes to the antiarrhythmic effects in human atrial muscle strips. The exact downstream mechanisms of NP and Sac/Val's antiarrhythmic properties are yet to be studied. All in all, an antiarrhythmic effect of Sac/Val based on effects of cellular Ca^2+^ handling seems to be likely. We have probed VASP phosphorylation in trabeculae treated with Sac/Val, as an indicator of increased PKG activity. However, VASP phosphorylation is highly time-dependent and rapidly decreases over time, no longer being significant only 1 h after treatment with ANP (22).

In light of the potential antiarrhythmic properties of Sac/Val on atrial tissue, ARNI treatment should be evaluated in high-risk patients with simultaneous HF and atrial fibrillation or atrial remodeling in future studies. Especially, in this patient group with high morbidity and mortality, it is likely that early ARNI treatment could exert major beneficial effects beyond the classically known ARNI effects by suppressing the induction of atrial arrhythmias and, therefore, maintaining atrial function.

## Conclusion

In this study, we could demonstrate that both BNP and Sac/Val exert beneficial effects on human atrial myocardium and that NEP expression in progressing HF with reduced EF is downregulated as part of an adaptive mechanism, and our data suggest that the favorable effects of Sac/Val are partly due to the increased concentration of NPs in the myocardium and that these effects are already achieved in therapeutic clinical doses.

### Limitations

For this study, we were working with human myocardial samples obtained from routine heart surgeries. As these human samples are not available on a large scale, we had to work with rather small sample sizes. Furthermore, all patients come with a unique combination of comorbidities and genetic variations. Therefore, we had to deal with a heterogeneous group in terms of underlying conditions, which, on the positive side, realistically reflects the clinical variation of patients. For the experiments with Sac and Val, we had to use DMSO as a solvent. More recently, DMSO was identified to influence human cellular processes and to exert toxicity already on low doses (44, 45). To account for these factors, we added DMSO to our control groups.

## Data Availability Statement

The original contributions presented in the study are included in the article/Supplementary Material, further inquiries can be directed to the corresponding author/s.

## Ethics Statement

The studies involving human participants were reviewed and approved by Local Ethics Committee of the Charité (EA2/167/15) and the Ethics Vote for the Biobank at the DHZB (EA4/028/12). The patients/participants provided their written informed consent to participate in this study.

## Author Contributions

FRH and UP conceived the study design and obtained patients informed consent. FRH obtained Ethics Committee approval and funding. UP collected the patients' clinical data and performed echocardiography analysis. FH, FB, CK, VF, and HG collected right atrial appendages from all patients. PD acquired and analyzed right atrial trabeculae function and assisted in molecular biology. KT contributed to acquisition and analysis of tissue preparation and right atrial trabeculae function. CK furthermore provided patient characteristics for molecular biology studies on NEP expression. DL provided methodological support and contributed to interpretation of the results. PW performed Western Blot and ELISA and interpretation of these results. UP and PD equally contributed to the analysis. FRH, UP, and PD drafted the manuscript and interpretation of all data. BP and FH provided valuable feedback to the manuscript draft. All authors contributed to the manuscript and approved the submitted version.

## Funding

This project was funded by a research grants from Novartis Deutschland GmbH. PD received an MD scholarship from the DZHK. FRH is supported by research grants from the German Research Foundation (DFG: CRC1470 and HE 7737/4-1).

## Conflict of Interest

The authors declare that the research was conducted in the absence of any commercial or financial relationships that could be construed as a potential conflict of interest.

## Publisher's Note

All claims expressed in this article are solely those of the authors and do not necessarily represent those of their affiliated organizations, or those of the publisher, the editors and the reviewers. Any product that may be evaluated in this article, or claim that may be made by its manufacturer, is not guaranteed or endorsed by the publisher.
