# Supplementary material for: Effects of BNP and Sacubitrilat/Valsartan on Atrial Functional Reserve and Arrhythmogenesis in Human Myocardium
Source: Front Cardiovasc Med. 2022 Jul 5;9:859014. doi: 10.3389/fcvm.2022.859014 (PMC9294287; doi:10.3389/fcvm.2022.859014)
Supplement: Supplementary file 1 [file Data_Sheet_1.pdf]

Supplemental Material

Functional Experiments

Biopsies of the RA appendage (size range: 1-2cm x 0,5-2 cm) were collected as part of the standard surgical procedure for patients undergoing open heart surgery with extracorporal circulation. Patients with active malignancy, congenital heart disease and endocarditis were not enrolled in our study and one patients was excluded due to reported chronical right coronary artery occlusion. Immediately after excision, the tissue was transferred into a cardioplegic solution containing: 100 mM NaCl, 10 mM KCl, 1.2 mM KH_2PO_4, 5 mM $MgSO_4$, 5 mM MOPS, 50 mM Taurine, 20 mM glucose and 30 mM 2,3-butanedione monoxime, equilibrated to a pH of 7.4. The tissue was then transported to the laboratories and processed into atrial trabeculae for functional measurements and tissue-samples for molecularbiology. Trabeculae were excised using microsurgical scissors and forceps. Human atrial muscle strips with a diameter of <1mm were preferentially isolated, as the risk of central ischemia increases with rising muscle diameter; muscle strips with a diameter <0.65 mm were excluded After the end of the experiments (trabeculae) or right after preparation (tissue-samples), all tissue was snapfrozen with liquid nitrogen and stored at -80° up until further analysis. After a resting phase of 10 min in cardioplegic solution, RA trabeculae were transferred into an isometric force transducer system, (Myostation-Intact muscle analysis system, MyoTronic UG, Heidelberg, Germany), hooked up in a loop system and superfused with normal Tyrode (136 mM NaCl, 4 mM KCl, 1 mM $MgCl_2$, 10 mM HEPES, 20 mM glucose and 0.2 mM $CaCl_2$) at 37°C. $CaCl_2$ concentration was gradually increased up until 2.5 mM over the course of 10 minutes and RA trabeculae were electrically paced with rectangular pulses (5 ms) field stimulation at 1 Hz and then gradually prestretched to their maximal force of contraction. Maximal isometric twitch force was reached when the increase of muscle prestretch did not cause greater force of contraction. Each contractile cycle was recorded and transferred to the graphical user interface

MyoDat (MyoTronic, Heidelberg, Germany) on a computer assigned to each setup. Analysis of systolic and diastolic functional parameters was performed with the program MyoViewer (MyoTronic UG, Heidelberg, Germany. Furthermore, the occurrence of mechanical evidence for arrhythmias (=aftercontractions) was determined.

VASP Western Blot

Atrial muscle strips from n=5 patients (control strips and strips treated with LBQ/Val) were homogenized at 4°C in lysis buffer with the following composition: 20 mM Tris-HCl, pH 7.4, 137 mM NaCl, 10% glycerol, 1% NP 40, 20 mM NaF, 1 mM sodium pyrophosphate, 50 mM β-glycerophosphate, 10 mM EDTA, 1 mM EGTA, 1 mM PMSF, 4 µg/ml aprotinin, 4 µg/ml pepstatin A, and 4 µg/ml leupeptin. For Western blotting 35 µg of tissues homogenates were run on on 4-12% Bis-Tris polyacrylamide gels and then transferred to nitrocellulose membranes. Non-specific binding was blocked with 5% dried milk in Tris-buffered saline (pH 7.4) containing 0.1% Tween-20. Membranes were probed with anti-Phospho-Ser239 VASP (1/500) or anti-Phospho-Ser157 VASP (1/750)(both Cell Signaling) and anti-VASP (1/750) (immunoGlobe) overnight at 4°C. Anti-rabbit IgG linked with IRDye 800CW and anti-mouse IgG linked with 680RD (both LI-COR) were used as a secondary antibody. The signal was detected with Odyssey CLx System. The band intensities were determined by Image Studio software (LI-COR).

NEP ELISA

The NEP levels were determined in LV and RA from n=10 patients with end-stage HF. Tissue was analyzed by the ELISA kit (EHMME, Thermo Scientific) according to the manufacturer’s instruction. Briefly, the frozen tissue samples were homogenized at 4°C in lysis buffer with the following composition: 20 mM Tris-HCl, pH 7.4, 137 mM NaCl, 1% NP 40, 20 mM NaF, 1 mM sodium pyrophosphate, 50 mM β-glycerophosphate, 10 mM EDTA, 1 mM EGTA, 1 mM

PMSF, 4 µg/ml aprotinin, 4 µg/ml pepstatin A, 4 µg/ml leupeptin. and centrifuged at 12,000 ×g for 10 min to remove debris. The total protein concentration in supernatants was determined via Pierce BCA assay (Thermo Scientific). Samples were then diluted 1:5 and assessed in duplicate The concentration (ng/ml) was normalized to total protein content (ng/mg total protein).

A

Patient characteristics	
Age	52 (45-56)
Sex (male)	50% (5/10)
BMI	23 (21-31)
NYHA III/IV	80% 8/10
Heart failure	100% 10/10
Arterial hypertension	30% 3/10
Dyslipidemia	60% 6/10
Adipositas	30% 3/10
Diabetes mellitus	30% 3/10
Coronary artery disease	60% 6/10
Atrial Fibrillation	50% 5/10
COPD	20% 2/10
Chronic kidney disease	30% 3/10
Post CABG/AVR/MVR	40% 4/10
Device (ICD, PM)	80% 8/10
PAH	40% 4/10

Type of surgery	
Heart transplantation	70% 7/10
Assist device implantation	30% 3/10

Medication	
ACE Inhibitors/ AT1-RB	70% 7/10
Beta-Blocker	80% 8/10
Diuretics	80% 8/10
MRI	80% 8/10
ARNI	10% 1/10
Digitoxin	10% 1/10
Calcium Antagonists	10% 1/10
Amiodaron	30% 3/10
Statine	60% 6/10
Oral antidiabetic	10% 1/10
Insulin	0

Main diagnosis	
Ischaemic CM	40% 4/10
Dilatative CM	30% 3/10
Myocarditis	20% 2/10
Restrictive CM	10% 1/10

B

Echo data		
LVEF (%)	20 (15-26)	N=10
LVEDD (mm)	68 (60-82)	N=10
IVSd (mm)	12 (10-13)	N=10
PWd (mm)	10 (8-12)	N=10
LA volume biplan (ml)	106 (78-122)	N=10
LAVI (ml/m2)	51 (40-61)	N=10
LA diameter (mm)	46 (41-50)	N=10
LA emptying fraction (norm >37%)	27 (18-34)	N=10
LA strain (>23% norm)	11 (7-16)	N=9
RVEF (%)	37 (33-41)	N=10
RA area(cm2)	23 (17-33)	N=10
RA diameter (mm)	44 (30-52)	N=10
RA emptying fraction (norm >37%)	31 (29-38)	N=8
RA strain reservoir+conduit	19 (16-40)	N=6
E/É	0	N=0
TAPSE (mm)	14 (13-16)	N=9
RVEDD (mm)	40 (33-49)	N=10
sPAP (mmHg)	38 (35-44)	N=4

Supplementary Table 1: Patient characteristics for RA and LV NEP expression. Data is presented as ‘median (IQR)’ or ‘percent (n patients/all patients)’. (a) represents clinical characteristics, (b) represents results from echocardiographic assessment

Abbreviations: CABG: coronary artery bypass graft, AVR: aortic valve replacement/repair; MVR: mitral valve replacement/repair; PAH: pulmonary artery hypertension; MRI: mineralocorticoid receptor inhibitor; ARNIs: angiotensin receptor-neprilysin inhibitor; CM: cardiomyopathy; LV: left ventricle, LA: left atrium, RA: right atrium

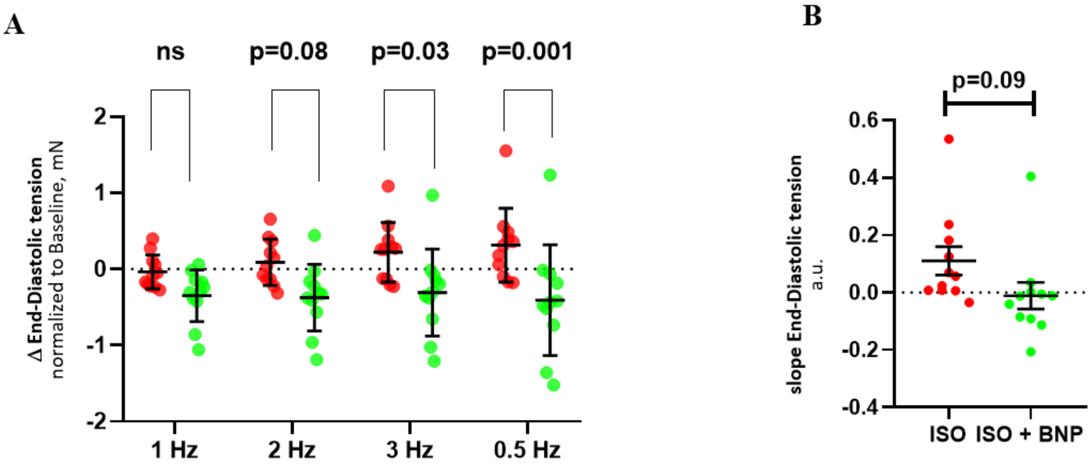

Supplement Figure 1: Effect of BNP on diastolic tension

Development of diastolic tension during varying stimulation frequencies is shown in (A), and corresponding diastolic tension slope is shown in (B). Data is shown as mean±SEM; each data point represents one muscle strip. *ISO: isoproterenol, BNP: brain natriuretic peptide*

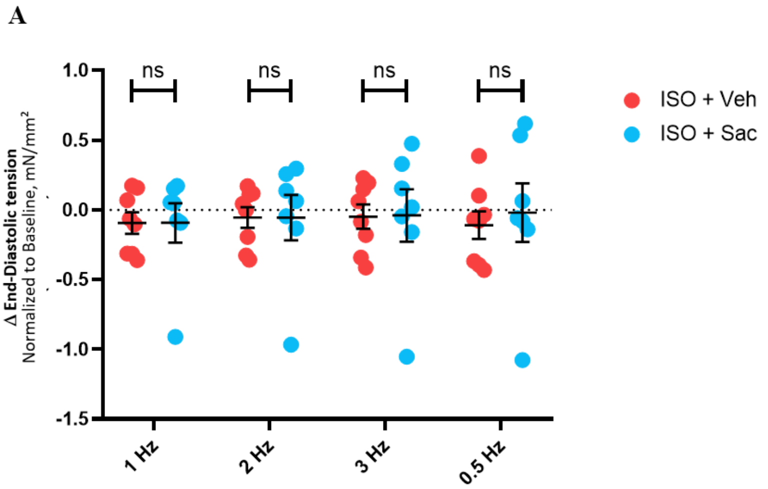

Supplement Figure 2: Effect of Sac on diastolic tension

The development of diastolic tension during varying stimulation frequencies in muscle strips pretreated with Sac or a vehicle and additional ISO treatment is shown in (A). Data is shown as mean±SEM; each data point represents one muscle strip. *Veh: vehicle (DSMO); Sac: sacubitrilat*

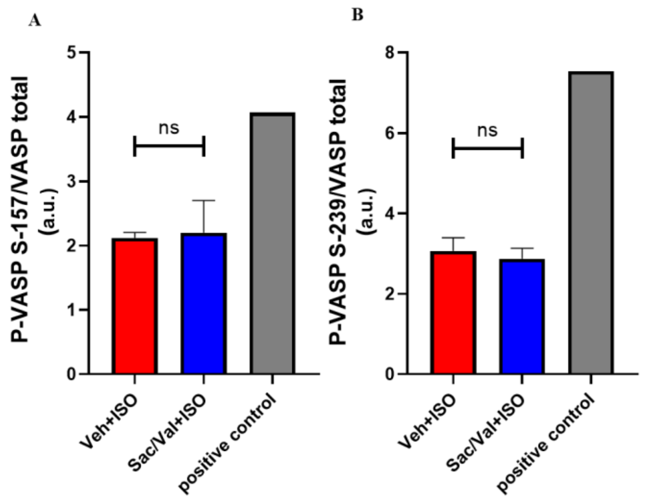

Supplement Figure 3: Effects of Sac/Val on VASP phosphorylation

The effect of Sac/Val on VASP Serine-157 (A) and VASP Serine-239 Phosphorylation is shown in this table. *Veh: vehicle (DSMO); Sac: sacubitrilat*; Val: valsartan

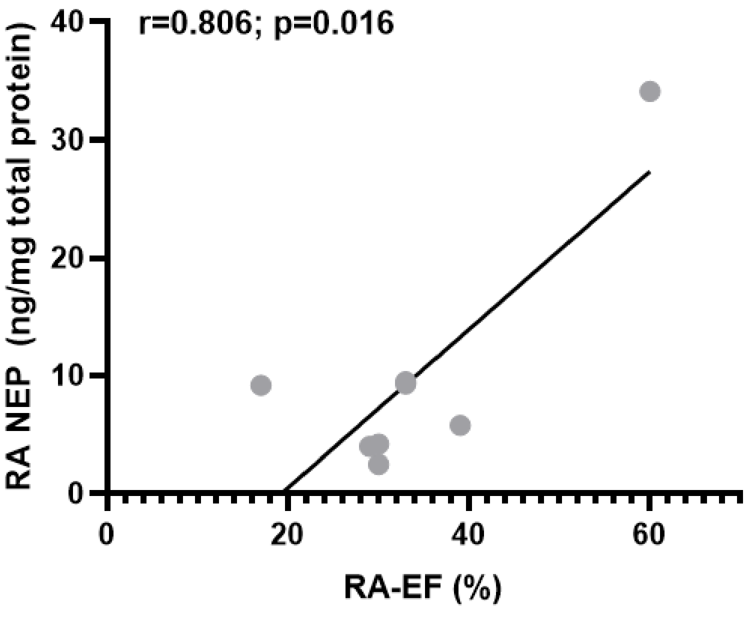

Supplement Figure 4: Correlation of right atrial NEP expression and right atrial EF

Each point represents data from one patient. *RA: right atrium; NEP: neprilysin; EF: ejection fraction.*
